# A Rare Case of Sjogren’s Syndrome-Related Recurrent Pleural Effusion

**DOI:** 10.7759/cureus.20685

**Published:** 2021-12-25

**Authors:** Dina Alnabwani, Shakumar Patel, Vraj Patel, Veera Jayasree Latha Bommu, Jia Hong Chen, Shawn Keating, Pramil Cheriyath

**Affiliations:** 1 Internal Medicine, Hackensack Meridian Ocean Medical Center, Brick, USA; 2 Internal Medicine, Hackensack Meridian Ocean Medical Center, Kadapa, USA; 3 Internal Medicine, Hackensack Meridian Health Ocean Medical Center, Brick, USA

**Keywords:** sicca syndrome, connective tissue disorder (ctd), b cell lymphoma, autoimmune, lymphoproliferation, sjogren syndrome, pleural effusion

## Abstract

Sjogren’s syndrome (SS) is a systemic autoimmune disease marked by lymphocyte infiltration of the exocrine glands and a variety of systemic symptoms. The wide range of prevalence reported in different studies is due to the fact that SS respiratory symptoms are polymorphic and vary in severity. Some 9%-20% of patients with SS have clinically severe lung impairment. Pleural effusion in SS has an etiology that is unknown. It is thought to be caused by CD4+ T cells secreting cytokines that cause B lymphocytes to generate autoantibodies. High beta-2-microglobulin, which is secreted by lymphocytic tissue particularly in pulmonary SS, is another sign of lymphoproliferation in lung tissue. Our patient had recurrent pleural effusion due to lymphoproliferation in the lung as a result of SS.

## Introduction

Sjogren’s syndrome (SS) is a systemic autoimmune illness characterized by lymphocyte infiltration in exocrine glands, particularly the salivary and lacrimal glands, causing xerophthalmia and xerostomia. Women are more likely to be affected (female to male ratio of 9:1), and symptoms usually appear in middle life (40-50 years old). Chronic obstructive pulmonary disease (COPD) (in 10% of cases), bronchiectasis (in 8% of cases), and interstitial lung disease (in 8% of cases) are the most common complications of lung involvement (in 5%). Pleural effusion is exceedingly uncommon, occurring in less than 1% of patients with SS, and is primarily seen in Europe and Japan [1]. Because there is no known cure for SS, treatment usually focuses on symptom relief and the prevention of complications such as opportunistic infections due to the lack of saliva and tears, increased risk of hematological malignancy, depression, anxiety, sleep disturbances, and inflammation-related disorders of the lungs, pericardia, liver, kidneys, nerves, and central nervous system. We report a rare case of recurrent pleural effusion in a 69-year-old female with a known history of SS. 

## Case presentation

A 69-year-old female presented for evaluation of shortness of breath. She started to experience chest tightness which was aggravated with inspiration and was self-resolved. She denied any chest pain, palpitation, diaphoresis, or fever. A review of the system was positive for sinus pain, cough, shortness of breath, palpitation, leg swelling, and headache. On admission, her vitals were blood pressure 162/84 mmHg, heart rate 101 bpm, respiratory rate 20/min, with 96% O2 saturation. Physical exam showed obese ill-appearing female with respiratory distress, tachycardic irregular rhythm with the presence of edema in lower extremities. She has a past history of hospital admissions for recurrent pleural effusion, left side video-assisted thoracoscopic surgery (VATS) with decortication and partial pleurectomy, possible trapped lung, recent oxygen use during the hospital stay. She also has a history of Sjogren’s syndrome (SS), fibromyalgia, thyroid cancer, atrial fibrillation, hypertension, and large B cell lymphoma. 

**Figure 1:**
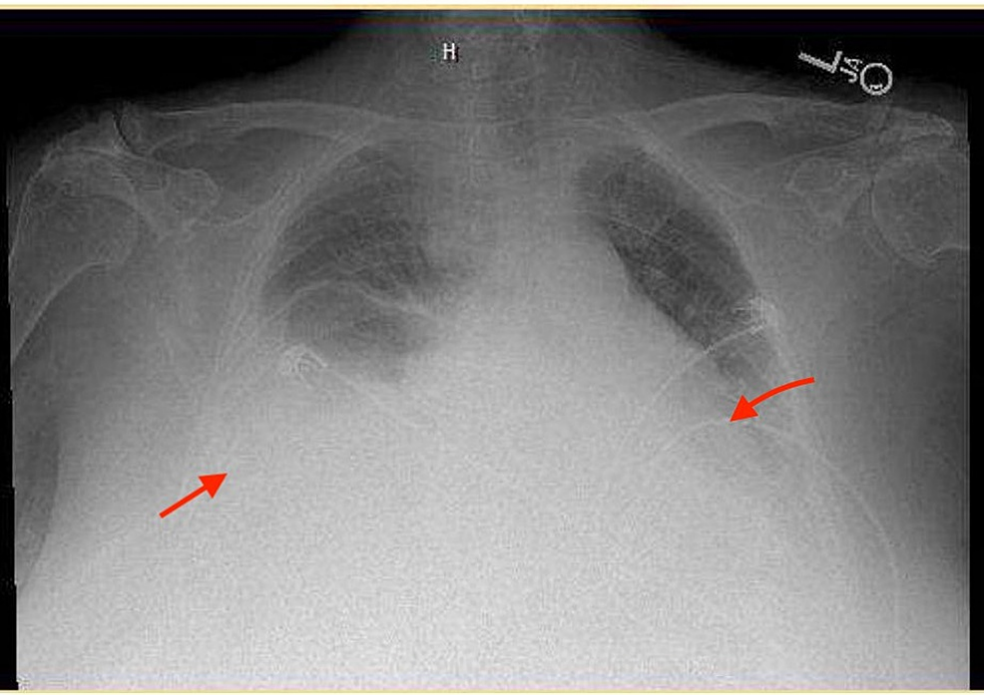
Chest X-ray PA view: moderate right pleural effusion, minimal left pleural effusion. PA, posteroanterior

**Figure 2:**
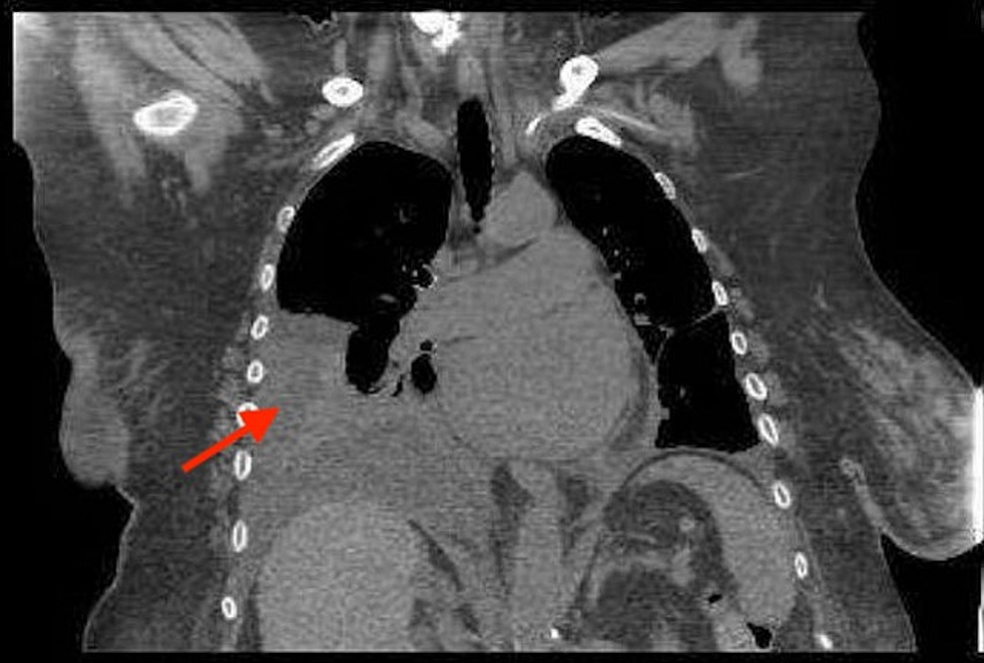
CT chest without contrast coronal view showing moderate right pleural effusion (red arrow).

**Figure 3:**
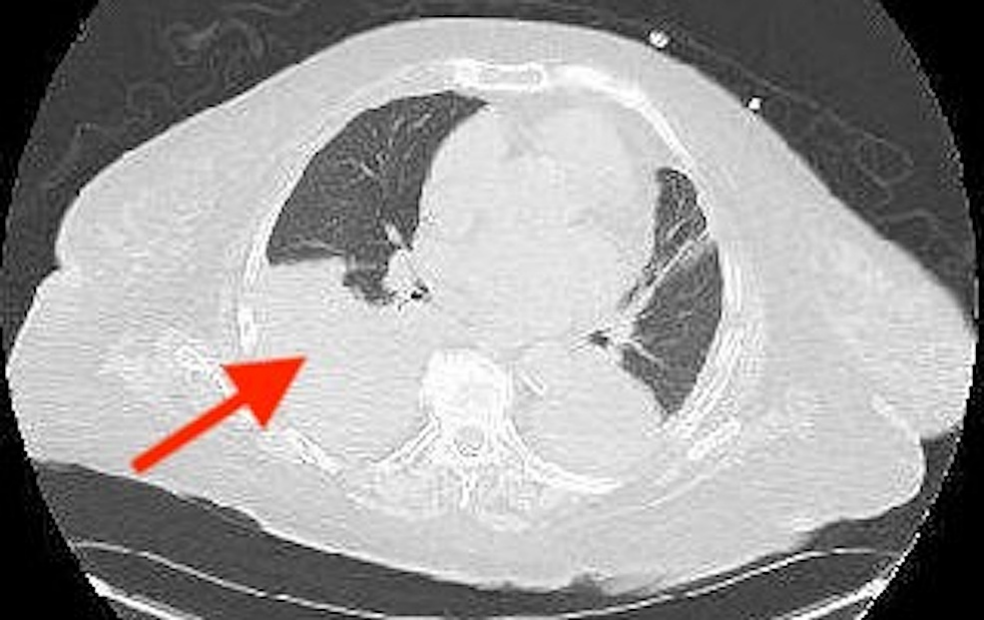
CT chest without contrast axial view showing moderate right pleural effusion (red arrow).

Initial troponin I was unremarkable. Her laboratory report showed hemoglobin of 11.7 g/dL (12-15.5 g/dL), lymphocytes 0.6, glucose 141 mg/dL (80-140 mg/dL), alkaline phosphatase (ALP) 144 IU/L (44-147 IU/L), and carbon dioxide (CO2) 34 mEq/L (23-29 mEq/L). Chest X-ray posteroanterior (PA) view showed moderate right pleural effusion and minimal left pleural effusion (Figure 1). CT chest without contrast showed consolidation-like changes at both lower lobes with moderate right pleural effusion (Figures 2-3). Pleural fluid analysis revealed a protein of 3.4 g/dL, lactate dehydrogenase (LDH) of 3920 IU/L, albumin of 1.9 g/dL, cell count: polymorphonuclear cells (PMNs) 2%, lymphocytes 3%, other 95%, cytology was positive for malignancy, and culture fungus negative. Right pleural flow cytometry revealed monoclonal kappa light chain restricted B cells (78% of total cells), consistent with B cell lymphoproliferative disorder.

 

## Discussion

Sjogren’s syndrome is a systemic autoimmune illness characterized by exocrine gland lymphocyte infiltration and a variety of systemic symptoms. SS can be a standalone condition [primary Sjogren’s syndrome (pSS)] or it can be linked to other autoimmune diseases such as systemic lupus erythematosus (SLE), rheumatoid arthritis (RA), dermatomyositis, and systemic sclerosis (SSc) [2]. Tracheobronchial sicca and interstitial pulmonary fibrosis are the most common presentations of respiratory involvement in SS, while some cases are also complicated by pulmonary arterial hypertension, pulmonary lymphoma, pseudolymphoma, amyloidosis, and lymphocytic interstitial pneumonitis [3]. However, pleural effusion is an extremely rare complication of SS. A retrospective Chinese study by Dong-Fang Lin et al., consisting of 573 patients of primary SS reported an incidence of pleural effusion as 5.7% [4]. 

The SS respiratory signs are polymorphic and vary in severity, which explains the vast range of prevalence reported in different research. In SS, 9%-20% of people have clinically severe lung damage [5]. The annual incidence of respiratory symptoms is predicted to be 10% (3%) after one year following SS diagnosis and increases to 20% (4%) after five years [5].

The exact etiology of pleural effusion in pSS is not known. It is presumed to be due to cytokines released from CD4+ T lymphocytes which might activate B lymphocytes to produce autoantibodies. These autoantibodies are anti-Ro/SS-A, anti-La/SS-B, associated with pleuritis and other systemic tissue damage. Both increased lymphocytes and anti-SS-A/SS-B antibodies were observed in the pleural effusion [6]. T-cell receptor beta-chain variable region gene bias and local autoantibody production in the pleural effusion were reported by Kawamata et al. [7]. In the course of SS, a wide range of systemic symptoms might arise. After dry eye and dry mouth, fatigue is the most prevalent symptom in pSS, affecting 70%-80% of patients. The cause is unknown, and the etiology could be complicated. In roughly 30%-40% of patients with pSS, further systemic symptoms arise [8].

The predominant histological lesion of pSS is a progressive localized lymphocytic infiltration around the salivary and lacrimal ducts that spreads and replaces the physiological glandular epithelium, resulting in dry eyes and mouth. These lesions are shown to be infiltrated by mononuclear cells enriched in CD4+ T-cells. The development of pulmonary SS has been linked to a complex combination of genetic, environmental, and hormonal variables [9]. One of the pathogens implicated in the etiology of SS is human T-lymphotropic virus type 1 (HTLV-1). Anti-HTLV-1 antibodies are far more common in SS patients with airway disease than in those who do not [10]. High beta-2-microglobulin, which is secreted by lymphocytic tissue and elevated in serum from patients with pSS, particularly pulmonary SS, is another sign of lymphoproliferation in lung tissue [11-12]. 

Our patients' chest X-ray posteroanterior (PA) view showed moderate right pleural effusion and minimal left pleural effusion (Figure 1). While CT chest without contrast showed consolidation-like changes at both lower lobes with moderate right pleural effusion (Figures 2-3). Pleural fluid analysis revealed a protein of 3.4 g/dL, lactate dehydrogenase (LDH) of 3920 IU/L, albumin of 1.9, cell count: PMNs 2%, lymphocytes 3%, other 95%, cytology was positive for malignancy, and culture fungus negative. Right pleural flow cytometry revealed monoclonal kappa light chain restricted B cells (78% of total cells), consistent with B-cell lymphoproliferative disorder which was thought to be the underlying reason for her recurrent pleural effusion associated with SS.

Patients with pSS may experience dyspnea (62%), cough (54%), sputum production (14%), chest discomfort (11%), and/or fever (7%). Anti-Ro/SSA antibodies are present in nearly three-quarters of patients, while anti-La/SS-B antibodies are present in one-third [13]. Pleural effusion is uncommon in SS, occurring in less than 1% of patients and predominantly documented in Japan, with a few cases reported in Europe. Pleural effusion in SS should always raise the potential of an overlap syndrome with SLE or RA (secondary SS) because pleural effusion is more common in the latter connective tissue disorders (CTDs) than in SS. Pleural infection or lymphoma is the most common differential diagnosis [8]. Bilateral pleural effusions are more common. It is usually exudative, with a lymphocytic cell count in the majority. Increased pleural and/or serum levels of rheumatoid factor, anti-SSA/Ro, anti-SSB/La, and immune complexes may also be seen in the pleural fluid study. Complement levels in the pleural and/or serum may also be reduced. Pleural thickness has been found to be more frequently related to recurrent pneumonia and atelectasis in patients with SS [8].

Pleural effusion in pSS is confirmed after excluding all the other possible etiologies. Pleural fluid analysis shows elevated lymphocyte count along with anti-SS-A and anti-SS-B antibodies. Lymphocyte‐abundant bronchoalveolar lavage (BAL) and lymphocyte‐infiltration of pleura evidenced on pleural biopsy provide evidence to diagnose SS-related pleural effusion [14]. Above mentioned findings and rapid response to steroid treatment are helpful to diagnose SS‐related pleural effusion. Treatment of SS-related pleural effusion seems to show that controlling the systemic inflammation with the help of corticosteroids showed good response, as in classical primary SS [15]. However, Mycobacterium infection should be excluded before the initiation of steroids.

## Conclusions

Despite the fact that pleural effusion is a rare indication of primary SS, it should be explored in individuals who have this condition and whose etiology is unknown. Nonetheless, a precise diagnosis necessitates not only specific biochemical markers but also a long period of follow-up to rule out other possible causes.
